# A novel injectable hydrogel containing polyetheretherketone for bone regeneration in the craniofacial region

**DOI:** 10.1038/s41598-022-23708-6

**Published:** 2023-01-17

**Authors:** Mahdieh Alipour, Marjan Ghorbani, Masume Johari khatoonabad, Marziyeh Aghazadeh

**Affiliations:** 1 Dental and Periodontal Research Center, Faculty of Dentistry, Tabriz University of Medical Sciences, Tabriz, Iran; 2 Nutrition Research Center, Tabriz University of Medical Sciences, Tabriz, Iran; 3 Department of Oral Radiology, Faculty of Dentistry, Tabriz University of Medical Sciences, Tabriz, Iran; 4 Stem Cell Research Center, Tabriz University of Medical Sciences, Tabriz, Iran; 5 Department of Oral Medicine, Faculty of Dentistry, Tabriz University of Medical Sciences, Daneshgah St., Golgasht St., Tabriz, 5166614711 Iran

**Keywords:** Stem cells, Medical research

## Abstract

Polyetheretherketone (PEEK) is an organic material introduced as an alternative for titanium implants. Injectable hydrogels are the most promising approach for bone regeneration in the oral cavity to fill the defects with irregular shapes and contours conservatively. In the current study, injectable Aldehyde-cellulose nanocrystalline/silk fibroin (ADCNCs/SF) hydrogels containing PEEK were synthesized, and their bone regeneration capacity was evaluated. Structure, intermolecular interaction, and the reaction between the components were assessed in hydrogel structure. The cytocompatibility of the fabricated scaffolds was evaluated on human dental pulp stem cells (hDPSCs). Moreover, the osteoinduction capacity of ADCNCs/SF/PEEK hydrogels on hDPSCs was evaluated using Real-time PCR, Western blot, Alizarin red staining and ALP activity. Bone formation in critical-size defects in rats’ cranial was assessed histologically and radiographically. The results confirmed the successful fabrication of the hydrogel and its osteogenic induction ability on hDPSCs. Furthermore, in in vivo phase, bone formation was significantly higher in ADCNCs/SF/PEEK group. Hence, the enhanced bone regeneration in response to PEEK-loaded hydrogels suggested its potential for regenerating bone loss in the craniofacial region, explicitly surrounding the dental implants.

## Introduction

Over the past few decades, bone tissue engineering provided a promising alternative for reconstructing bone defects in the craniofacial region^1–3^. Bone defects in this area occurs due to congenital abnormalities, infections, and subsequent bone resorption through trauma, tumor resection, and tooth extraction^4,5^. These defects significantly affect patients’ quality of life and should be treated to regain maxillofacial function and esthetic^6,7^.

Dynamic bone structures exhibit remarkable regenerative abilities in reconstructing defects smaller than critical size. Critical size defects do not have self-healing ability and need additional reconstruction intervention^8^. Conventional approaches for treating these kind of bone defects, such as autologous/allogenic bone grafts and metallic prosthesis, are limited by the morbidity and shortage of donor sites, the possibility of resorptions, and complicated fabricating and shaping to fill irregular defects^9,10^. Due to these limitations, bone tissue engineering approaches with biodegradable and biocompatible 3D polymeric hydrogels can be considered as a potential candidate for increasing the effectiveness of treatment protocols by improving stem cells proliferation and differentiation. Similar properties of hydrogels to native bone extracellular matrix (ECM) and delivering osteogenic factors are the main traits that make a hydrogel suitable for bone regeneration. Another essential characteristic that should be considered is the injectability of these hydrogels, which provides a wide range of advantages compared to prefabricated hydrogels^11–13^. These injectable hydrogels demonstrate outstanding support for infiltration, attachment, proliferation, and differentiation of stem cells when fabricated with suitable polymers and substances. Moreover, the easy handling, minimally invasive administration, and fulfilling the irregular shaped bone defects are the advantageous of these injectable hydrogels in clinical application besides to the patients’ convenience^14–18^.

Various biomaterials have been applied to reconstruct damaged and lost bone tissue^19–21^. Polyetheretherketone (PEEK) is a promising organic and synthetic polymer with a semi-crystalline structure. It has become more popular due to its high biocapability, radiolucency, and similar elasticity to the natural bone compared to metallic materials such as titanium (Ti)^22–24^. Moreover, according to the reports, titanium implants and their alloys have shown metal ion release, osteolysis, allergenicity, and metal corrosion during reconstructing bone defects in the craniofacial region^25,26^. The application of PEEK in different substrates and materials for more than 40 years approves its great capacities for biomaterial application. As a high-performance polymer, PEEK features excellent chemical resistance, a high melting temperature of 340 °C, superior radiation and sterilization resistance, a high modulus of elasticity of 3.7 to 4.0 GPa, and high tensile strength of 103 MPa^27^. Among prosthetic materials, PEEK is widely used as a component due to its good heat stability and similar mechanical properties to natural bone. These properties help PEEK-based composites to promote bone regeneration and delay adjacent bone resorption^28^. A wide range of spinal implants have been made from PEEK, including cages, rods, and screws, that are designed to remain rigid while the bones gradually fuse together^29–32^. The most notable features of PEEK, which turns it into good material in the craniofacial region, include but are not limited to excellent mechanical properties, natural radiolucency, and reduced heat transforming^19,33^. Furthermore, there are several pieces of evidence regarding reduced osteolysis, increased bone formation and supporting initial mineralization around PEEK implants^19^. However, some studies showed that this substrate is not as bioactive as Ti; therefore, they suggested the incorporation of PEEK with other materials^22,34,35^.

Due to its significant characteristics, silk fibroin (SF) is an appropriate biopolymer for synthesizing hydrogels. However, this material indicates less mechanical strength, which could be solved by crosslinking with other materials^36^. Cellulose nanocrystals are natural polysaccharides with remarkable physicochemical properties, which turn this substance into a potent reinforcing agent for biomedical applications, especially bone tissue engineering^37^.

Although different studies demonstrate the notable characteristics of PEEK-based materials, there were no studies evaluating PEEK-containing injectable hydrogels with in-situ gelation behavior to enhance the osteogenic capacity of this material for bone regeneration. Therefore, in the current study, the Aldehyde-cellulose nanocrystalline/silk fibroin/ PEEK (ADCNCs/SF/PEEK) hydrogels were synthesized, and their osteogenesis capacity was evaluated both in vitro and in vivo.

The successful fabrication of this hydrogel and its porous structure was characterized by Fourier- transform infrared spectroscopy (FTIR), thermogravimetric analysis (TGA), and scanning electron microscopy (SEM). In in vitro phase, the cytocompatibility of the fabricated hydrogels was evaluated by 3’(3-[4,5-dimethylthiazol-2-yl]-2,5 diphenyl tetrazolium bromide) (MTT) assay on human dental pulp stem cells (hDPSCs). HDPSCs are easy-access multipotent stromal cells extracted from pulp tissue with high efficiency and low morbidity. They can be safely cryopreserved to be used in clinical trials. As osteoblasts, these cells can synthesize 3D woven bone tissue chips and synergistically differentiate into endotheliocytes and osteoblasts. These mesenchymal stem cells appear immune-privileged as they can be grafted into allogeneic tissues and exert anti-inflammatory effects^38^. The osteogenic differentiation of these cells on fabricated hydrogels was also measured by Alizarin red staining, Alkaline phosphatase activity (ALP), Real-time PCR, and Western blot for the related markers. Furthermore, the newly formed bone in a critical-sized rat cranial defect was evaluated by histology and cone-beam computed tomography (CBCT).

## Results

### Hydrogel analysis

Average gelation times of ADCNCs/SF hydrogels with and without PEEK was (63.21 ± 6.35 s) and (83.10 ± 9.21 s) respectively which did not display a statistically significant difference.

**Figure 1:**
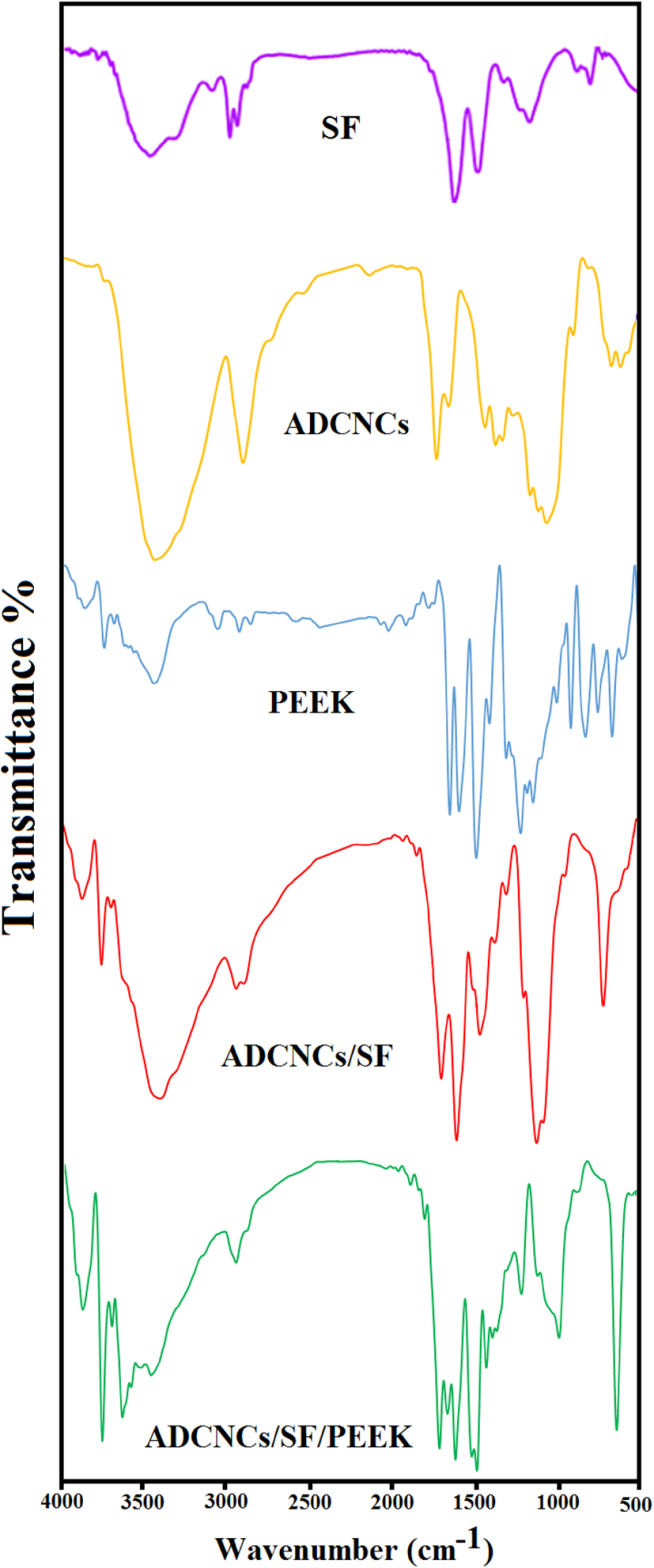
FTIR Spectra of ADCNCs, SF, PEEK, ADCNCs/SF, and ADCNCs/SF/PEEK.

FTIR spectra of ADCNCs, SF, PEEK, ADCNCs/SF, and ADCNCs/SF/PEEK were displayed in Fig. 1. The FTIR spectrum of silk fibroin showed strong absorption bands at 1540 cm^−1^, and 1244 cm^−1^ (amide II and III), 1660 cm^−1^ (amide I, CO, and CN stretching), 3300 cm^−1^ (NH stretching), respectively. The bands at 1500–1300 cm^−1^ and 1100–900 cm^−1^ could also be related to the CH bending and skeletal stretching regions, respectively. Moreover, the -gly-gly- sequence of the silk fibroin chain was observed by the band at 1016 cm^−1^. The FTIR spectrum of the PEEK revealed the asymmetric stretching vibration peak for R–O–R at 1217.06 cm^−1^, the aromatic ring framework vibration peak at 1593.49 cm^−1^, and 1485.73 cm^−1^, the stretching vibration peak for C=O at 1648 cm^-1^; and symmetric stretching vibration peak for R–CO–R at 925.29 cm^−1^. Moreover, the peak at 835.27 cm^−1^ and 765.07 cm^−1^ can be attributed to the bending vibration absorption peaks for C–H out of the benzene ring plane. The aromatic ring para-position substitution was observed at 836.92 cm^−1^. In the spectrum of ADCNCs, the symmetric vibration at ~ 1742 cm^−1^ can be corresponded to hemiacetal formation of free aldehyde groups of AD-CNCs. After preparing ADCNCs/SF hydrogel, a new absorption peak was observed at 1680 cm^−1^due to the chemical crosslinking between amino groups of SF and dialdehyde groups of ADCNCs. Furthermore, the presence of a new peak at 1648 cm^−1^ confirmed the successful incorporation of PEEK into the ADCNCs/SF scaffold.

**Figure 2:**
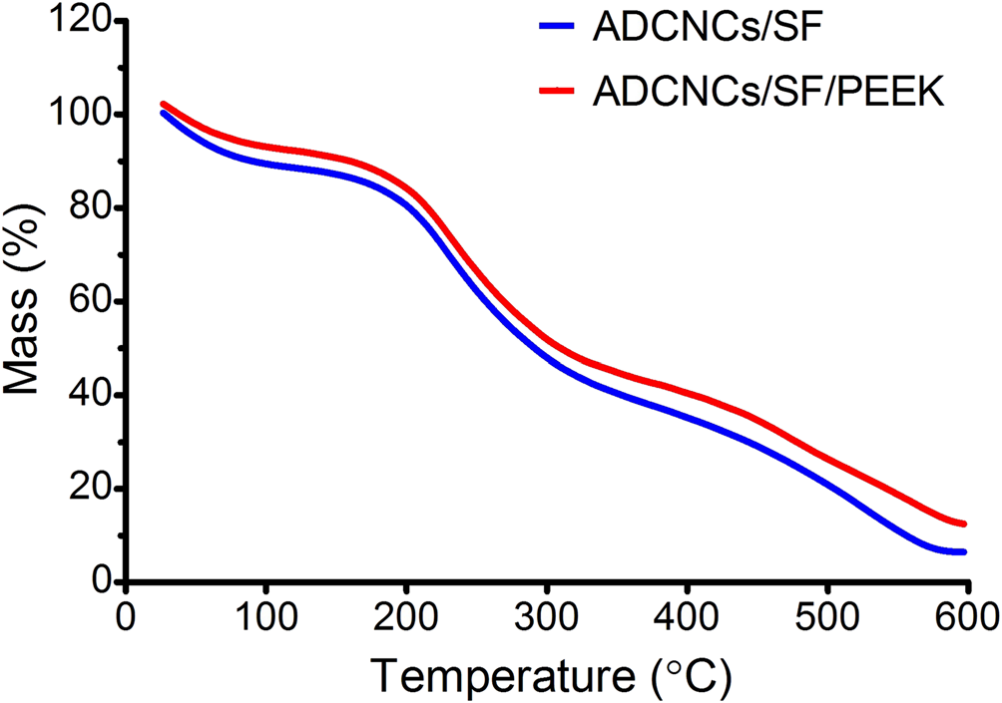
TGA curves of ADCNCs/SF and ADCNCs/SF/PEEK.

The results of thermogravimetric analysis of ADCNCs/SF and ADCNCs/SF/PEEK hydrogels are shown in Fig. 2. TGA curves of the hydrogels show a weight loss in three stages. The first stage (30–210 °C) is related to the loss of absorbed and bound water, about 6% loss in weight for ADCNCs/SF and ADCNCs/SF/PEEK hydrogels. The second stage, 210–310 °C, is due to the degradation of ADCNCs and SF with a loss in weight of 37%. The third weight loss step corresponds to the chain’s disassociation or rearrangement. According to the previous literature, adding mineral compounds such as PEEK increases the residual weight of thermal degradation, representing improved thermal stability^39–41^.

**Figure. 3:**
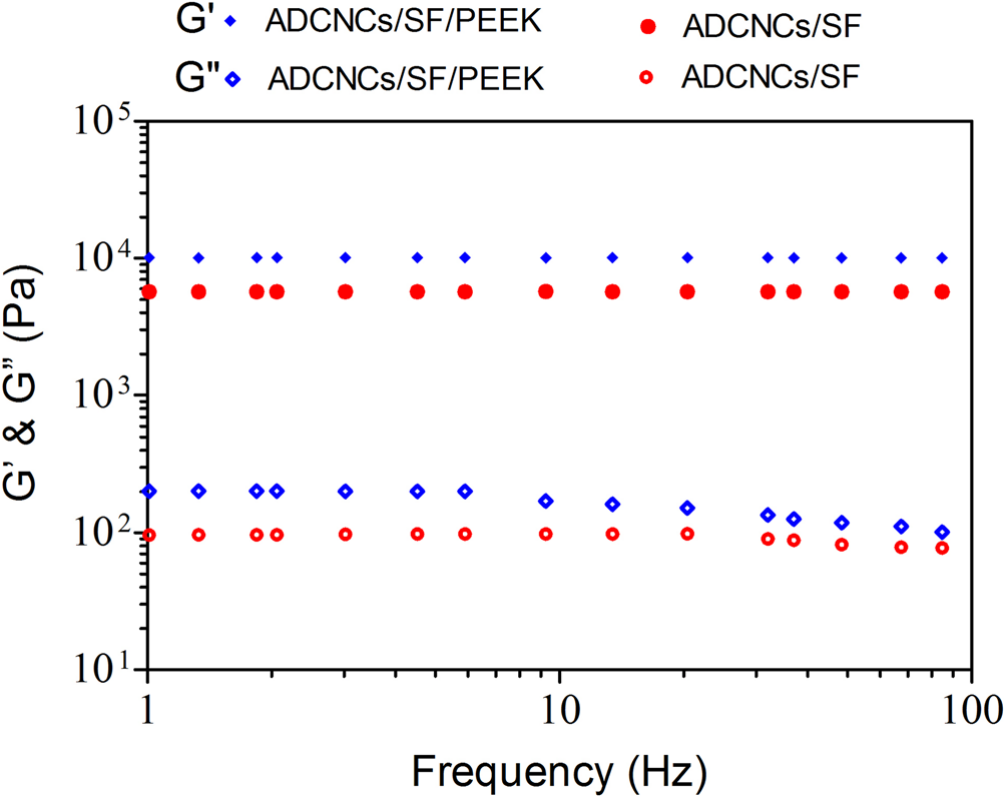
Frequency sweep of ADCNCs/SF and ADCNCs/SF/PEEK hydrogels.

The rheological behaviors of developed hydrogels were conducted by oscillatory rheology. As a function of angular frequency, the association of storage (G′) and loss modulus (G″) of the hydrogels was characterized in Fig. 3. The rheological behaviors mainly be influenced by the covalent bonds between the amino groups of SF and aldehyde functionality, electrostatic and hydrogen bonding between SF and ADCNCs, and the reinforcement in the network^42^. The results showed that G″ was lesser than G′ for the SF/ADCNCs hydrogels containing PEEK, which claims a stable crosslinked network enhanced quickly with the PEEK content. Moreover, the good interfacial compatibility between SF, ADCNCs, and PEEK was observed because SF/ADCNCs/PEEK offered a 1.1-fold higher magnitude of G′ compared with SF/ADCNCs.

### In vitro degradation and swelling behavior studies

**Figure 4:**
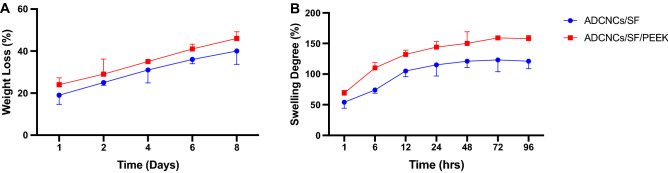
(**A**) In vitro degradation profile of ADCNCs/SF and ADCNCs/SF/PEEK. (**B**) Swelling degree of ADCNCs/SF and ADCNCs/SF/PEEK hydrogels. Data are expressed as mean ± standard deviation (*n* = 3).

The results of the degradation study showed the reduction of degradation rate in the ADCNCs/SF/PEEK hydrogels due to the decrease in the mobility of network chains, leading to the low penetration of water molecules in the hydrogel structure. Therefore, ADCNCs/SF/PEEK hydrogels had a slower degradation rate than ADCNCs/SF hydrogels (Fig. 4A).

As noted in previous studies, the swelling properties of hydrogels mainly depend on the hydrophilic ability of the functional groups, crystallinity, and mechanical strength^43^. In this regard, the results indicated that the swelling of hydrogel composites had been reached to equilibrium swelling of the hydrogel composites 12 h after being immersed in PBS (Fig. 4B). This finding suggested the fast-swelling characteristics of hydrogel. The swelling ratio of the ADCNCs/SF hydrogel was statistically higher than that of ADCNCs/SF/PEEK hydrogels, confirming that the addition of PEEK as the reinforcement material influenced the crystallinity of the matrix. On the other hand, the swelling ratio decreased as the PEEK was incorporated because of its high crystallinity and hydrophobicity nature.

### SEM images

**Figure 5:**
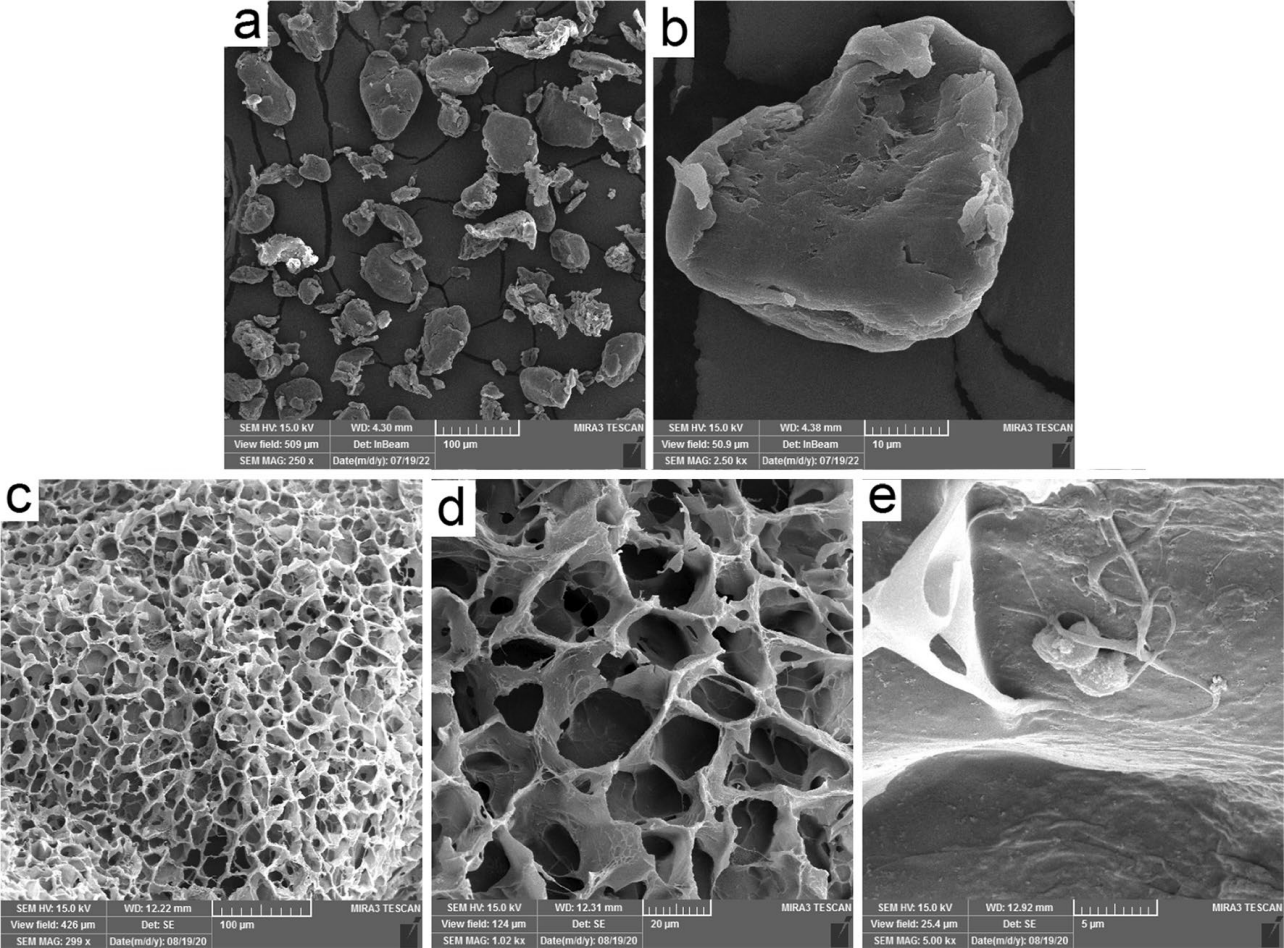
SEM image. (**a**) PEEK particles at low magnification, (**b**) PEEK particles at high magnification, (**c** and **d**) ADCNCs/SF/PEEK hydrogel; (**e**) Human Dental Pulp Stem Cells on fabricated hydrogels.

The morphology of PEEK powder in a low magnification image showed that the PEEK particles have near-spherical shape (Fig. 5a). The high magnification image revealed that the surface of particles was relatively rough (Fig. 5b). The composite presented interconnected porous structures (Fig. 5c). The morphology of ADCNCs/SF/PEEK scaffolds with higher magnification is shown in Fig. 5d. The SEM evaluations demonstrated homogenous porous scaffold. Moreover, the adhesion of hDPSCs on the scaffold structure was revealed by SEM (Fig. 5e).

### MTT assay

**Figure 6:**
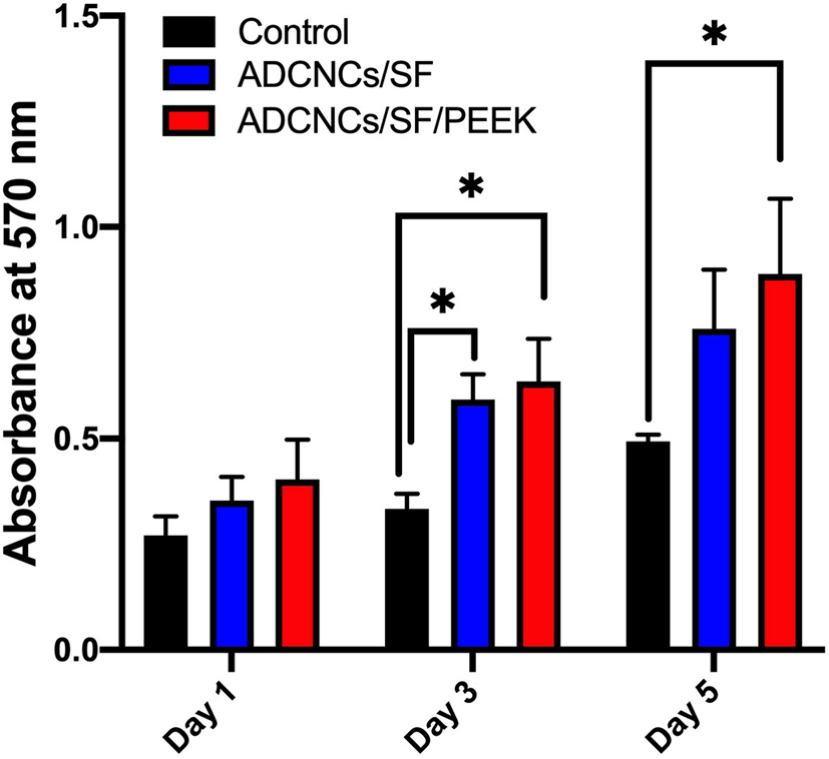
MTT assay indicated the cytocompatibility of synthesized scaffolds. The proliferation of hDPSCs seeded on hydrogels was increased over time. This proliferation were raised significantly on day 3 and 5 after seeding of stem cells on hydrogels. Control: DPSCs without facing hydrogels, ADCNCs/SF: DPSCS seeded on hydrogel without PEEK, ADCNCs/SF/PEEK: DPSCS seeded on hydrogel containing PEEK (*P < 0.05).

The proliferation of the hDPSCs was determined by MTT assay 1, 3, and 5 days after seeding on ADCNCs/SF and ADCNCs/SF/PEEK hydrogels. Compared to the control group (cells without hydrogels), the viability of hDPSCs was increased in all the groups (Fig. 6). Moreover, the proliferation of hDPSCs seeded on hydrogels was increased over time. In other words, the proliferation of hDPSCs were raised significantly in a time-dependent manner. Generally, 3 and 5 days after seeding of stem cells on ADCNCs/SF/PEEK hydrogels, a significant increase in OD value was observed.

### Alizarin red staining and alkaline phosphatase activity in hDPSCs

**Figure 7:**
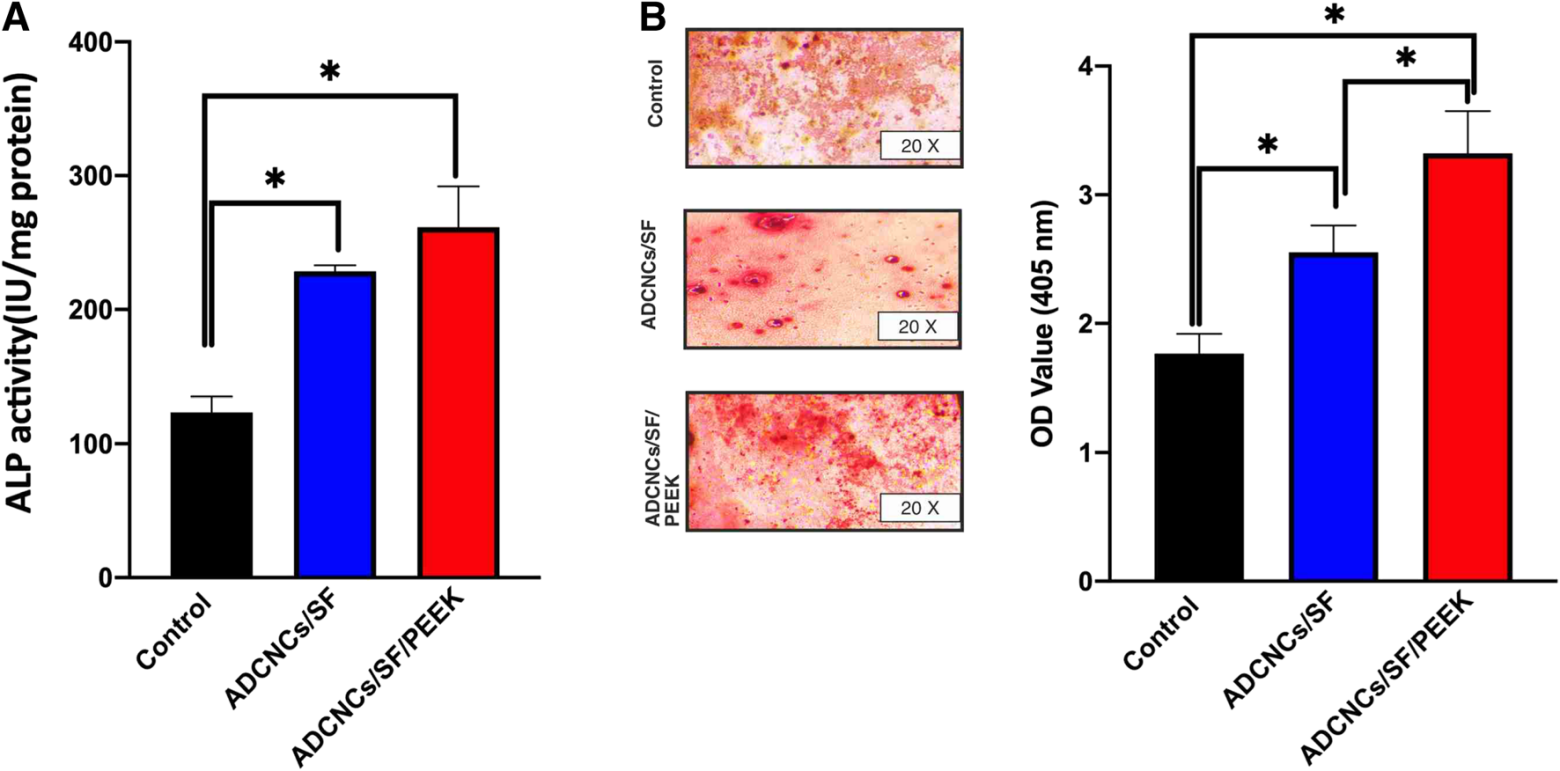
(**A**) Alkaline phosphatase activity increased significantly in both fabricated hydrogels compared with the control group (**B**) Alizarin red staining was performed to detect the calcium accumulation in hDPSCs. Compared with control, the red color intensity was significantly higher in ADCNCs/SF and ADCNCs/SF/PEEK hydrogels. Moreover, adding PEEK into the scaffold backbone showed drastically high mineralization compared to the control group. Control: DPSCs without facing hydrogels, ADCNCs/SF: DPSCS seeded on hydrogel without PEEK, ADCNCs/SF/PEEK: DPSCs seeded on hydrogel containing PEEK (*P < 0.05).

The ALP enzyme activity was measured seven days after seeding of hDPSCs on hydrogels. According to the results, the expression of this enzyme was significantly increased in ADCNCs/SF/PEEK groups compared to ADCNCs/SF and control groups (*P* < 0.00). ALP activity in experimental groups was 123.428, 228.5933, and 261.47 (IU/mg protein) in control, ADCNCs/SF, and ADCNCs/SF/PEEK groups, respectively (Fig. 7.A).

The calcium deposition of hDPSCs on injectable hydrogel containing PEEK particles was significantly increased, which was detected by the red stains in the Alizarin Red S staining assay (Fig. 7B).

### Expression of osteogenic genes and proteins in hDPSCs

**Figure 8:**
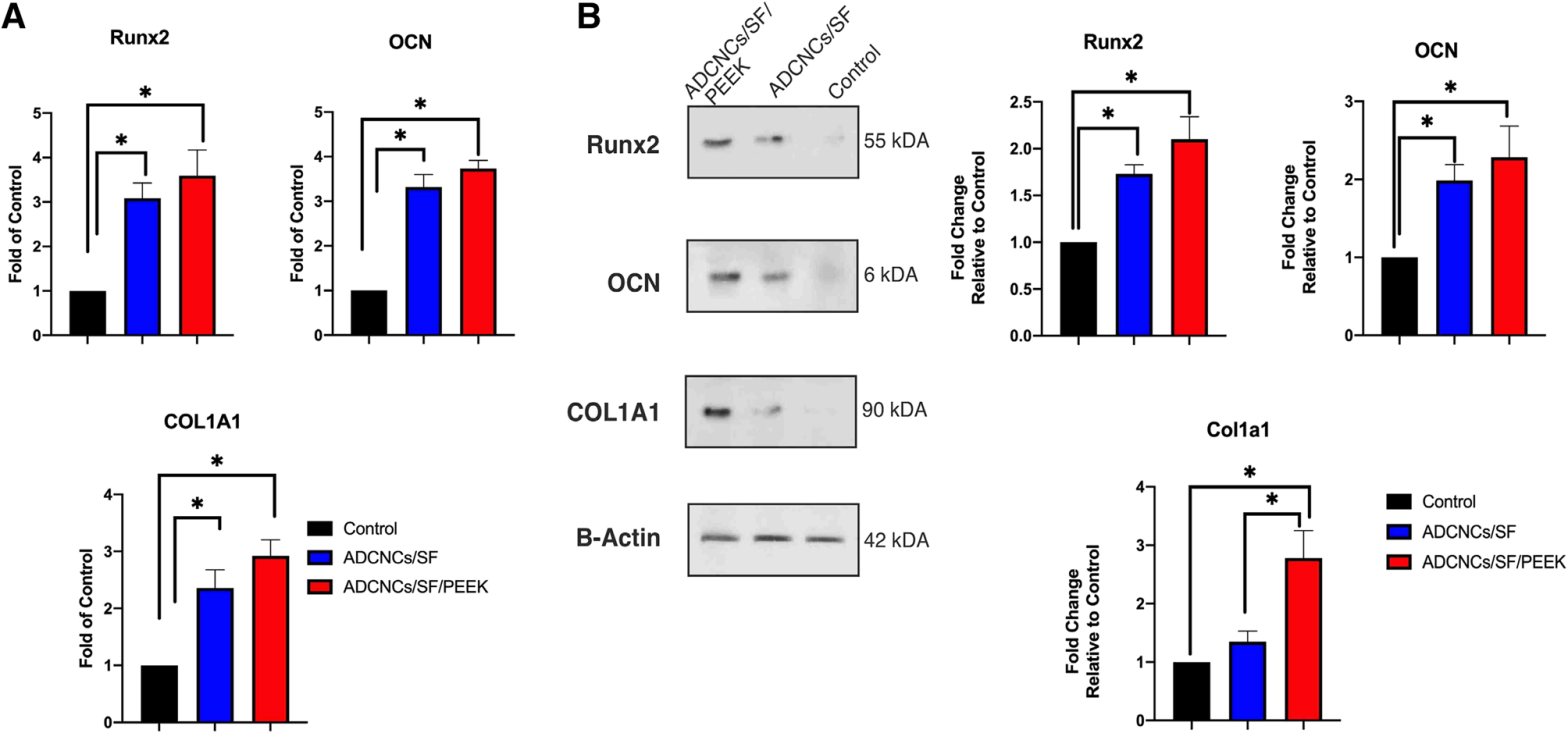
(**A)** Expression levels of Runx2, OCN, and COL1A1 genes in hDPSCs seeded on synthesized hydrogels after 14 days increased drastically. (**B)** Western blotting evaluated the secretion of the same factors in protein levels after 7 days. Seeding of hDPSCs on the synthesized hydrogels also increased the expression of evaluated osteogenic markers in protein levels. Control: DPSCs without hydrogels, ADCNCs/SF: DPSCs seeded on ADCNCs/SF hydrogel, ADCNCs/SF/PEEK: DPSCs seeded on hydrogel containing PEEK (*P < 0.05).

The influence of synthesized hydrogels on the osteogenesis of HDPSCs was analyzed by the expression of osteogenic markers, including Runx2, osteocalcin (OCN), and COL1α1, both in gene and protein levels. According to the results, the seeded cells on ADCNCs/SF/PEEK had higher mRNA expression levels (Fig. 8A). This difference was significant compared with ADCNCs/SF and control groups in all evaluated genes. Western blot analysis also revealed that ADCNCs/SF/PEEK component led to a markedly up-regulation in the expression of Runx2, COL1α1, and OCN proteins (Fig. 8B). As shown, the western blot results were consistent with the PCR data. The results demonstrated that the presence of PEEK particles in the hydrogel induced the expression of osteogenic genes and proteins in hDPSCs and promoted their differentiation to osteoblasts.

### CBCT analysis in the critical size calvarial defects

**Figure 9:**
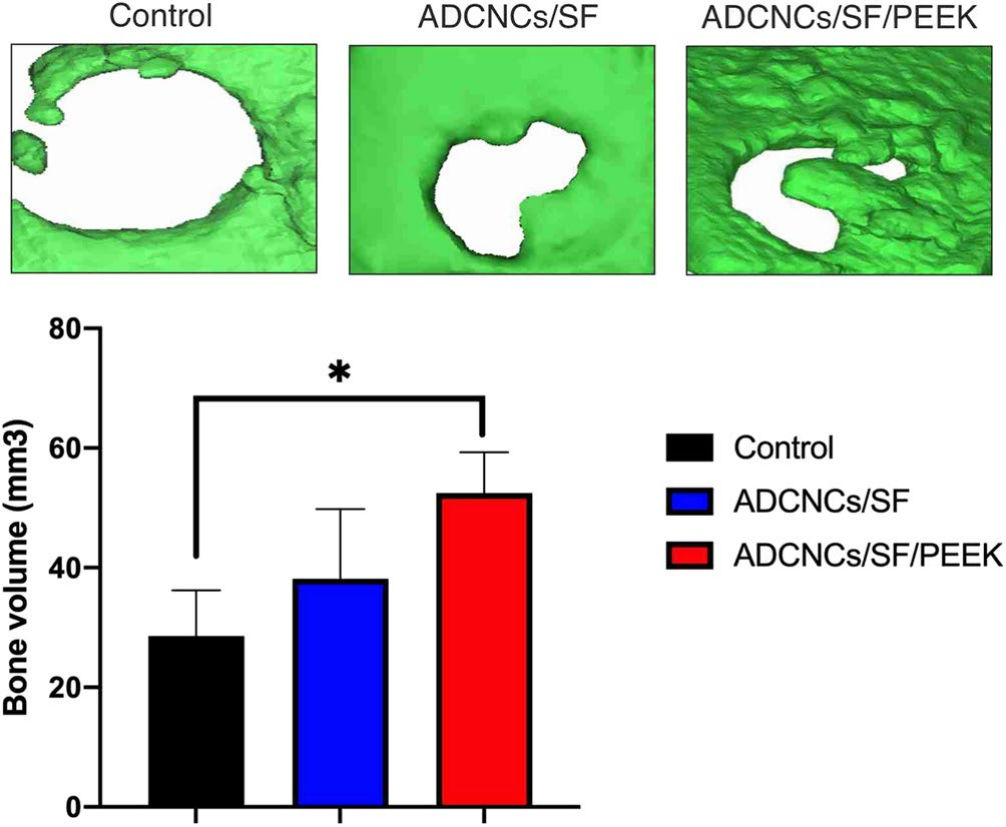
ADCNCs/SF/PEEK injectable hydrogels drastically increased bone formation after 8 weeks, as proved by CBCT. The almost full closure occurred in ADCNCs/SF/PEEK group, while ADCNCs/SF group caused less bone volume in the defect area. Bone volume (mm^3^) was measured in defects sites of all samples. The significant difference was between ADCNCs/SF/PEEK groups with control. Control: critical-sized bone defects without any treatment, ADCNCs/SF: critical-sized bone defects filled with hydrogel without PEEK, ADCNCs/SF/PEEK: critical-sized bone defects filled with the hydrogel containing PEEK (*P < 0.05).

CBCT analysis was performed to evaluate bone formation in the defect area 8 weeks after implantation of hydrogels. The total volume of bone formation in the critical size defects was 28.59 ± 7.5990 mm^3^, while these amounts were 38.15 ± 11.63 mm^3^ and 52.5 ± 7.8 mm^3^ in ADCNCs/SF and ADCNCs/SF/PEEK groups, respectively. The differences among studied groups were significantly higher in ADCNCs/SF/PEEK hydrogels compared with the control group (Fig. 9).

### Histological evaluation of bone formation in the calvarial defects

**Figure 10:**
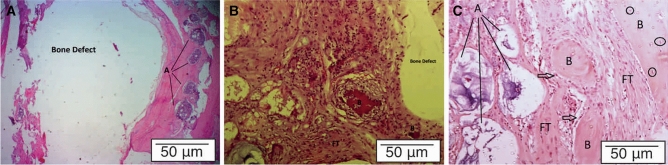
Histology of samples. (**A)** Critical-sized bone defects without treatment: A bone defect was identified in the central part of the section. Around the bone defect, matured bone containing the bone marrow (**A**) was detectable. (**B)** Critical-sized bone defects filled with hydrogel without PEEK (ADCNCs/SF): The major area of bone defects were filled with connective and fibrous tissue (FT). Bone spicules with central mineralization were observed in some parts (**B**). (**C)** Critical-sized bone defects filled with a hydrogel containing PEEK (ADCNCs/SF/PEEK): Various microcystic areas containing the primary phase of calcification (**A**) and several islands of new bone formation (**B**) were present in the healing area. The black arrows show osteoblasts around the bone spicules. The cavity was filled with regenerative tissue, including fibrous tissue (FT) and bone matrix. Matured bone containing osteoblasts (black circles) is obvious at the section's right top.

Eight weeks after surgery, no severe inflammatory or infectious reactions was observed in the groups. The hematoxylin and eosin (H&E) staining in the samples of the control group identified lamellar bone occupied by mature osteocytes and bone marrow around the defect. The bone defect's edge was obviously detectable in the tissue section, while a few osteoblastic cells were seen around the bone defect. This group had no significant bone regeneration (Fig. 10A). In the ADCNCs/SF group, new bone formation was started at the injection area in the bone defect. A few bone spicules with central mineralization surrounded by osteoblasts were presented around the defect area. Connective tissue containing fibroblasts and new blood vessels provided the healing process in different parts of this group. Some inflammatory cells were seen in the connective tissue; however, there were no obvious inflammation signs in the rats. Moderate bone regeneration was presented in this group, while most filled tissue was connective tissue (Fig. 10B). In the PEEK-containing group, the progress of greater new bone formation was noticeable. The defect area was filled with many micro-cystic areas containing the primary phase of bone formation. This group had no cell-free regions, and the whole defect was filled with regenerative tissue. Areas of primary and advanced stages of bone formation were presented. The slight formation of the bone matrix was identified. Several osteoblasts were detectable around the bone spicules. In some areas, the matrix was matured, and the deposition of minerals formed a new but matured bone containing osteoid cells. Connective tissue containing fibroblasts was seen, while there was no evidence of foreign body reactions (Fig. 10C).

## Discussions

During the past few decades, injectable hydrogels' application for reconstructing bone defects with irregular size and shape has attracted extensive attention. The current study's objective was to develop injectable hydrogel containing PEEK to regenerate critical size bone defects in cranial region. These surgically challenging defects must be carefully managed to reconstruct the lost area to achieve the desired function and esthetic. Injectable in situ forming hydrogels are promising approaches that can provide easy handling, facile and homogenous distribution of hydrogel in irregular and large defects before complete gelation of the hydrogel. Moreover, these materials are minimally invasive, which reduces the necessity for large incisions causing patient convenience, scar formation, and infection^44,45^.

Based on the results of the current study, injectable in situ forming hydrogels provide interconnected porous structures. This structure offers an appropriate surface for cell adhesion, nutrient and waste distribution^42^. Nontoxicity of fabricated hydrogels is another critical factor for developing biomaterials. The cytocompatibility of the developed hydrogel in this study was confirmed by MTT assay. Furthermore, the osteoinductive capacity of this hydrogel was observed both in in vitro and in vivo phases.

PEEK can be considered a primary candidate for the replacement of metallic implants due to its mechanical and chemical properties. However, it is reported as a bio-internet substance in some pieces of literature, which can be a roadblock to its biological and clinical applications^29,46,47^. Some studies demonstrated that this material could not induce as much proliferation as other similar materials such as titanium^19,48^. However, this shortage is trying to be covered by surface modulation. According to the evidences, cellular morphology and proliferation are directly affected by the surface roughness of materials^49^. Researchers have shown that moderate roughness of PEEK provided a significantly better cell attachment^50,51^. Moreover, some studies showed that topography plays a more significant role in cell attachment than chemical composition^51–53^. Therefore, cell adhesion and proliferation seem to be affected not only by the chemical composition but also by the topography of substrates. PEEK has shown proliferative effects in different cell lines^54^. Furthermore, a systematic review showed improved adhesion, proliferation, biocompatibility, and osteogenesis on the surface of PEEK implant materials^22^. In the current study, the fabricated ADCNCs/SF/PEEK hydrogels did not have any cytotoxicity on hDPSCs. HDPSCs on fabricated hydrogels provided better cell proliferation compared to control group. Moreover, adding PEEK on ADCNCs/SF backbone positively affected those cells. These results showed that the surface roughness of PEEK could be considered an advantage for cell proliferation. Similar results were achieved while the roughness of the surface increased in the presence of the PEEK^55^.

To evaluate the hDPSCs’ response to fabricated hydrogels, ALP, AlZ, real-time-PCR, and western blot tests were performed. ALP has been known as an early osto/odontogenic differentiation marker which is essential for the following mineralization. The underlying mechanism is related to hydrolyzing the phosphate ester with Alp as a transcription factor that can promote osteoblast differentiation^56,57^. The enhanced amounts of secreted phosphate level in ADCNCs/SF and ADCNCs/SF/PEEK groups compared with the control group demonstrated the higher secretion of this early differentiation marker. The evaluation of cellulose nanocrystal-based hydrogels showed an increased level of Alp activity in MC3T3- E1 cell lines, too^58,59^.

The Alizarin red staining was applied to evaluate the mineralization potential of developed hydrogels on hDPSCs. In this assay, the positive staining and high red color indicate deposition of calcium phosphate and mineralization^37^. PEEK-containing hydrogels demonstrated higher OD values than ADCNCs/SF and the control group, which suggested higher mineralization potential of ADCNCs/SF/PEEK hydrogels. Anionic matrices in the scaffold structure is bounded to the secreted calcium ions for formation of nucliation niches^42^. The other studies evaluated calcium deposition in silk fibroin and cellulose nanocrystals hydrogels on human bone marrow mesenchymal cells and suggested that this backbone elevated calcium deposition and cytoskeleton formation^37,60,61^.

Osteogenesis genes and protein expression were measured by real-time PCR and western blot techniques in the studied groups. The expression of three osteogenic specific markers (Runx2, OCN, and Col1α1) was measured in both gene and protein levels in hDPSCs exposed to fabricated hydrogels. The evaluation of these markers revealed a higher expression in cells with ADCNCS/SF and ADCNCS/SF/PEEK. These results suggested fabricated hydrogel's great osteogenic induction capacity for bone regeneration applications. There are two phases in natural bone formation: endochondral and intramembranous ossification. The former requires cartilage templates, while the latter happens due to mesenchymal condensation. The differentiation of mesenchymal stem cells to osteoblasts involves various transcription and signaling factors^36,62^. For instance, the expression of Runx2 is mandatory for differentiating mesenchymal stem cells into the osteoblastic phenotype^63^. Therefore, the higher amounts of this marker in ADCNCS /SF and ADCNCS/SF/PEEK compared with the control group suggested the higher osteogenic potential of hDPSCs after exposure to fabricated hydrogels.

The other factor expressed in immature mesenchymal stem cells and preosteoblasts is Col1α1. Meanwhile, osteocalcin is the other marker that is supposed to embed into the bone matrix and form the osteocytes^64,65^. Higher osteogenic potential of bone marrow mesenchymal stem cells was reported while they were seeded in chitosan/silk fibroin cellulose hydrogels. The authors cleared that hydrogel functional groups facilitate apatite formation in the extracellular matrix^60^. Liu et al*.* indicated osteoblastic differentiation capacity of bone marrow mesenchymal stem cells in 3D constructions of PEEK^66^. Some studies suggested the increased osteogenic capacity of PEEK while incorporated or modified by different substances^19,22,24,48^. The ADCNC/SF backbone greatly supports enhancing this material's biological properties. Therefore, the appropriate structure of fabricated hydrogels facilitated the osteogenic differentiation of DPSCs. This higher expression was even observed in protein levels in different studies using similar structures and backbone materials^37,67^.

In this study, we used rat models to determine bone formation in critical size bone defects. CBCT analysis was performed, and mimic software was used to evaluate the 3D reconstruction and bone volume. In addition, H&E staining were performed on bone samples. The newly formed bone was observed in both experimental groups containing the hydrogel. The healing procedure was superior in those groups compared with critical-sized bone defects without any treatment. These findings suggested the appropriate microenvironment of the hydrogels for bone regeneration. The positive effects of cellulose nano crystals-based biomaterial or regeneration of bone tissue were reported previously^37,60^. In Addition, it has been shown that silk fibroin-based scaffolds incorporation with ceramics and other bioactive molecules enhanced the expression of osteogenic markers and elevated bone regeneration in bony defects^68^. The current study incorporated silk fibroin with nanocrystalline cellulose and PEEK as an injectable in situ forming hydrogel. Although we did not evaluate the biomechanical properties in the newly formed bone, our results confirmed the desirable osteogenic induction for regeneration of critical-size cranial defects. Further investigation regarding optimizing this scaffold for load-bearing areas such as jaws considering more study groups, including PEEK-based orthopedic implants, are necessary for future studies.

## Methods and materials

### Materials

Silk cocoons from *Bombyx mori* (*B. Mori*) were obtained by the Silkworm Research Center (Gilan, Iran). Microcrystalline cellulose powder (MCC) was kindly donated from Zahravi Pharmaceutical Company (Iran). Sodium periodate (NaIO_4_), Dimethyl sulfoxide (DMSO), lithium bromide (LiBr), sodium carbonate (Na_2_CO_3_), and dialysis bag (MWCO = 3000 and 12,000 Da) were purchased from Sigma-Aldrich Co. (St Louis, MO, USA). PEEK was also purchased from Merck (Mean particle size 80 microns, GF75065755).

### Synthesis of injectable hydrogels

Silkworm cocoons were cut into pieces and then degummed for 30 min in boiling Na_2_CO_3_ solution to eliminate the sericin proteins. Then, the samples were rinsed thoroughly, deionized water several times and air-dried overnight. Next, the specimens were soaked in 9.3 M lithium bromide (1 g in 4 mL) for 4 h at 60 °C, then dialyzed (3 kDa MWCO) to remove LiBr for 4 days. The obtained solution was centrifuged to eliminate residual debris for further use.

The CNCs solution was prepared according to our previous work^69,70^. After that, the reaction of the CNC solution with NaIO_4_ solution (300 µg/mL) was performed in the dark condition for 8 h. To end the response, 600 µL ethylene glycol was added to the solution, and then the solution was dialyzed for further purification. The final product was freeze-dried to obtain the ADCNCs powder.

**Figure 11:**
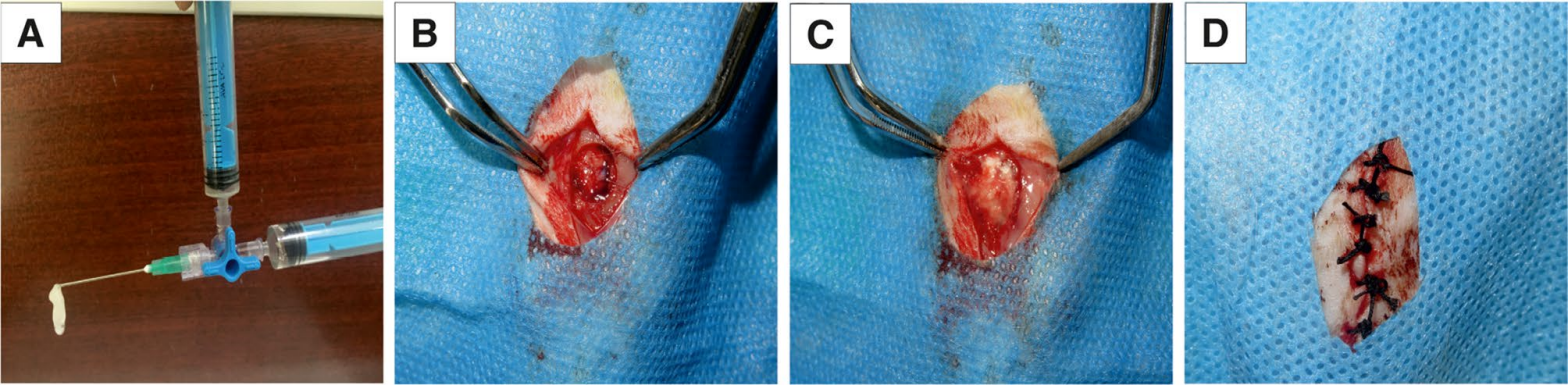
(**A)** Injectable ADCNCs/SF/PEEK hydrogel. (**B**) Critical size bone defect (8 mm) in rat cranial (**C)** ADCNCs/SF/PEEK hydrogel in created defect (**D)** Incision closure.

For this purpose, the prepared silk fibroin (~ 7%) and ADCNCs (~ 0.5% wt) solutions were transferred into two barrels and then injected into a mold, followed by keeping for 30 min to obtain the hydrogel (Fig. 11.A). The hydrogel was prepared using a double-barrel syringe with a gauge needle (21 G). For the hydrogel containing PEEK, the powder (10% wt) was added to the ADCNCs solution.

We calculated the aldehyde content to evaluate the oxidation degree of ADCNCs^71,72^. First, 2 mL of hydroxylamine hydrochloride solution (0.35 M) was added to 1 mL ADCNCs solution (pH = 5.0) and then stirred for 8 h at 55 °C. The feeding values of NaOH solution (0.5 M) were recorded as Vc and Vb for the titration of ADCNCs and CNCs. The molecular weight of ADCNCs is about 162 g/moL. Then, the aldehyde content was estimated by the following Eq:

$$ {\text{Aldehyde}}\;{\text{content}}\left( {\text{\% }} \right) = \frac{{\left( {Vc - Vb} \right) \times M{\text{NaOH}} }}{{\left( {m/Mw} \right)}} \times 100 $$

where m is the dry weight (g) of the ADCNCs sample.

### Characterizations of developed hydrogels

This study used the TGA (TGA/SDTA 851/Mettlear Toledo, Spain) device under nitrogen atmosphere (20 mL/min). The temperature range was from 30 °C to 600 °C at a heating rate of 10 °C/min. In compressed KBr pellets, the FTIR spectra of hydrogels were recorded with a resolution (4.0 cm^−1^) and 16 scans per minute by Bruker Tensor 27. The wavenumber range was set from 400–4000 cm^-1^. The inverted tube test was considered to calculate the gelation time.

The analysis of the frequency sweep test was completed with a range of frequencies from 1 to 100 rad/s and a regular strain of *ɤ* = 0.01. The dynamic viscosity, storage modulus (G′), and loss modulus (G″) were assessed.

The hydrogels were soaked in 20 mL of phosphate-buffered saline (PBS, pH = 7.4). Then, the samples were placed in a shaking incubator at 37 °C. After predetermined time intervals (1, 2, 4, 6, and 8), pieces were removed from the medium and dried under a vacuum. Degradation was quantified using the following Eq:

$$ {\text{WL }}\left( {\text{\% }} \right) = \frac{{{\text{W}}_{i} { }{-}{\text{ W}}_{f} }}{{{\text{W}}_{f} }} \times 100 $$

where Wi is the initial dry polymer mass, and Wf is the dry polymer mass at a time.

To assess the swelling ability of hydrogels, the specified samples were dipped into deionized water at 37 °C. In summary, at first, the dry weight of the hydrogels was measured (Wd), and then the swollen hydrogels were weighed after 1, 6, 12, 24, 48, 72, and 96 h (Ws). The swelling degree (SD) was recorded according to the Eq:

$$ {\text{SD }}\left( {\text{\% }} \right) = \frac{{{\text{W}}_{2} { }{-}{\text{ W}}_{1} }}{{{\text{W}}_{1} }} \times 100 $$

### Culture of hDPSCs on fabricated hydrogels

HDPSCs were purchased from Shahid Beheshti University and cultured on in High-content glucose Dulbecco's Modified Eagle Medium (DMEM/HG; Cat No: 31600083; Gibco; USA) containing %10 fetal bovine serum (FBS; Gibco) and 1% penicillin/streptomycin (Gibco, USA). After reaching to 80% confluency, cells were trypsinized (Gibco, Singapore) and seeded on the sterilized scaffolds for in vitro tests.

### Scanning electron microscopy (SEM) imaging

The surface morphology and structure of ADCNCS/SF/PEEK scaffolds with and without hDPSCs were evaluated by SEM three days after seeding. Before assessing, hDPSCs were fixed in 2.5% glutaraldehyde on the scaffolds as described recently^73^. After fixation, the hydrogels containing hDPSCs were dehydrated using a graded series of alcohol concentrations (50, 70, 90, and 100%)^74^. Afterward, scaffolds with and without stem cells were cut into three specimens and coated with a thick gold layer. To characterize these samples, FE-SEM 1430 vp (MIRA3 FEG-SEM—Tescan, Czech) was applied.

### MTT assay

The effects of synthesized scaffolds on hDPSCs were assessed by MTT assay. Briefly, 5 × 10^3^ cells were seeded on ADCNCs/SF and ADCNCS/SF/PEEK scaffolds in the 96 well plates. hDPSCs were cultured without scaffolds on the polystyrene surface of three wells in the same plate and were considered as a control group. After 1, 3, and 5 days, 50 μL of 3-(4,5-Dimethylthiazol-2-yl-2, 5-diphenyltetrazolium bromide) (Invitrogen, Carlsbad, CA, USA) solution (5 mg/mL) were added to the medium and after incubation for 4 h at 37 °C and 5% CO_2_; the medium was replaced by 100 μL DMSO and the color change of the purple formazan crystals solution was measured by a microplate reader (BioTek, USA) in the wavelength of 570 nm.

### Calcium deposition and alkaline phosphates activity

The calcium accumulation of hDPSCs seeded on ADCNCS/SF, and ADCNCS/SF/PEEK hydrogels were measured using Alizarin Red Staining to determine the mineralization of these cells. 5 × 10^5^ cells were seeded on the synthesized hydrogels, which were placed in 6 well plates as follows; three wells were filled with ADCNCS/SF hydrogels, three wells were filled with ADCNCS/SF/PEEK, and three wells contained hDPSCs without scaffolds, which was considered as the control group. After fourteen days, the cells were fixed with 2% paraformaldehyde (Sigma-Aldrich Co.) and washed with PBS. Then Cells were stained with 40 mM alizarin red (pH 4.2, Sigma-Aldrich Co.) for 40 min in the dark at room temperature. Finally, the wells were washed with distilled water three times and left to dry. The culture plates were photographed under an optical microscope to show mineralized nodules that appeared with a dark red center and light red peripheral area. For quantitative analysis, after adding 10% acetic acid solution for 30 min and shaking, 10% ammonium hydroxide solution was added to neutralize the reaction. The color intensity was determined using an ELISA reader (BioTek, USA) at 405 nm.

Alkaline phosphatase activity of seeded hDPSCs on synthesized hydrogels was determined based on manufacturer instruction (ALP assay kit, Pars Azmoon, Iran). Similar plates were prepared in the same condition as the ARS test. Seven days after cell seeding, the samples were washed twice with PBS and lysed in alkaline lysis buffer. After 45 min incubation, the concentration of P-nitrophenol was measured at 405 nm, and the results were reported as IU/mg protein.

### Evaluation of osteogenic-related markers by real-time PCR and western blot

1 × 10^6^ hDPSCs were cultured on the synthesized scaffolds, which were placed on the six well plates. After fourteen days, total RNA was extracted according to the manufacture instruction by Ambion TRIzol buffer (Cat No: 15596–026, Invitrogen, USA), and the quality of obtained RNA was determined with Nanodrop (Thermo Scientific, Waltham, MA, USA). Then, 1 μg of total RNA was used for cDNA synthesizing by a cDNA synthesis kit (Cat No: YT4500). The expression level of three genes for osteogenic differentiation was evaluated by specific primers, including Runx2, OCN, and COL1A1 (Table1). The β-actin gene was used as a housekeeping gene for normalization. The expression was measured by an RT-PCR system (LightCycler 96). Each data was repeated in three separate experiments and three times. Data analysis was performed by the Pfaffl method^75^.

**Table 1:** Sequences and melting temperature of primers.

Gene	Forward primer (5´ → 3´)	Reverse primer (5´ → 3´)	Tm
Runx2	CTCACTGCCTCTCACTTGCC	CTGTACACACATCTCCTCCC	65
OCN	TGTGTGAGCTCAATCCGGACT	CCTGGAGAGGAGCAGAACTGG	61
COL1A1	CAAGAGGAAGGCCAAGTCGAG	AGATCACGTCATCGCACAACA	59
B-Actin	AGTGTGACGTTGACATCCGT	TGCTAGGAGCCAGAGCAGTA	60

The immunoblotting assay was conducted to evaluate the level of expression of osteogenic proteins in hDPSCs seeded on ADCNCS/SF and ADCNCS/SF/PEEK hydrogels. HDPSCs were seeded without scaffolds considered as a control group. This test was performed according to standard protocols described in our previous study^76^. Briefly, after seven days, the cells were lysed in ice-cold cell lysis buffer solution (NaCl, NP-40, and Tris–HCl), including cocktail enzyme inhibitors. The solutions were sonicated and then centrifuged at 14,000 g for 20 min. The supernatant was analyzed for total protein contents by the Picodrop spectrophotometer system (Model No: PICOPET01, Serial No. 000212/1) and resolved by the SDS-PAGE method. The following primary antibody solution was added to samples and then was incubated overnight at 4 °C; Runx2 (RUNX2 (F-2), Cat No: sc-390351, Santa Cruz Biotechnology, Inc.), collagen type Ι alpha Ι (COL1α1 (3G3), Cat No: sc-293182, Santa Cruz Biotechnology, Inc.), osteocalcin (OCN (FL-100), Cat No: sc-30044, Santa Cruz Biotechnology, Inc.), and β-Actin (Cat No: sc-47778, Santa Cruz Biotechnology, Inc.). The samples were incubated with secondary HRP-conjugated anti-IgG antibody (Cat No: sc-2357, Santa Cruz Biotechnology, Inc.) for 1 h at room temperature. ECL plus solution kit (BioRad) was used to detect the immunoreactive blots. Visualizing the reactive proteins on the blots process was performed according to the manufacturer's instructions. This experiment was performed in triplicate.

### The surgical procedure in rat calvarial bone defects

Twelve mature Wistar rats, 8 weeks old and weighing 3500–400 g, were entered into the current study and randomly divided into three groups. Each group contained four rats, which were kept single in pathogen-free boxes for seven days to be adapted to the condition of the animal house. This condition included the temperature of 22 ± 5 °C, the humidity of 50–60%, and a cycle of dark/light for 12 h. All the rats had access to standard rat chow and water in the same amount and condition.

To evaluate the osteogenesis effect of synthesized hydrogels, one defect of 8 mm in diameter was created in the calvarial of each rat (Fig. 11B). For anesthesia, a ketamine (Rotexmedica, Trittau, Germany) /xylazine (Alfasan, Netherland) mixture (45/10 mg/kg) was injected intramuscularly. The calvarial area was shaved and disinfected with povidone-iodine. After that, an approximately 25 mm sharp incision was made by surgical blade #13. Prichard Periosteal Elevator was used to retract the tissues. Then an eight mm defect was created by the trephine bur of the dental implant kit (DASK, Dentium Advanced Sinus Kit, South Korea) while the surgical site was cooled by sterile saline. The created defects in four rats were filled by ADCNCS/SF, while the other four rates received ADCNCS/SF/PEEK hydrogels (Fig. 11C). The defect sites in remained four rats did not fill with any material and were considered the control group. The incisions were sutured using nonabsorbable 3/0 USP surgical black braided silk (HURTEB Medical Devices, Tehran, Iran) (Fig. 11D). For postoperative pain, the subcutaneous injection of Piroxicam (Exir, Tehran, Iran) was performed for each rat immediately after surgery and 24 h later. After rats became active, they were transferred to their boxes and received food and water, as mentioned. The surgery sites were covered with Gentamicin skin ointment to prevent infection. The sutures were removed seven days after surgery.

After eight weeks, rats were sacrificed by overdosing on pentobarbital (100 mg/kg), and the defect areas were removed from the calvarial of each rat with 2 mm extra safe margins. After the collection of bone samples, the carcasses were discarded by burial.

The bone pieces were washed with a phosphate buffer saline (PBS) to remove the attached surrounding tissue and fixed in 10% neutral buffered formalin (Pars Chemie, Tehran, Iran). New bone formation was analyzed using a radiology (CBCT) and histology (H&E) evaluation.

### CBCT assay

After sacrificing rats and harvesting the bony segments, the samples were scanned with Cone Beam Computed Scan (CBCT, NewTom VGi, Verona, Italy). The direction of the cone was placed parallel to the coronal surface of bone defects as described previously^77^. To create 3D reconstruction, the analysis was performed with Mimics Medical 21.0 (Materialise, Leuven, Belgium), and the total volume of bone formation was measured. Briefly, DICOM files were uploaded to the software, and for reconstruction, the lower and upper thresholds ranged between 0 and 700 Hounsfield units. The total bone volume was measured in the cylindrical region (8 mm × 1 mm). Four defect models were calculated for each group, and data were reported as mean ± SD.

### Histological examinations

Following the CBCT analysis, all samples were decalcified. In this process, 3% nitric acid (7697-37-2, Sigma, USA) was used for decalcification^78^. After decalcification, the specimens were bisected and dehydrated by the gradient of ethanol solutions in a tissue processor (MeyMed, DS 2080/H, Tehran, Iran). The dehydrated samples were embedded in paraffin wax blocks (HistoWax, SCILAB, UK). The samples were sectioned into 5 μm histological slides using a rotary microtome (DID SABZ, DS 4055, Urmia, Iran), then transferred to glass slides and glued with mounting medium (05-BMHM100, Bio Mount HM, Milano, Italy). Hematoxylin (PadtanTeb, Tehran, Iran) and Eosin (CARLO ERBA), were carried out to observe new bone formation under the light microscope (Olympus).

### Statistical analysis

Statistical analyses were performed using Prism software (version 8.0, GraphPad, San Diego, CA, USA). The Kolmogorov–Smirnov test analyzed the normality and homogeneity of the data distribution. The continuous values with normally distributed were reported as mean ± SD and analyzed by Student's t-test, one-way ANOVA, and Tukey post hoc analysis. *P*-value < 0.05 was considered statistically significant. All experiments were carried out in triplicates.

### Ethical approval

All experiments of the current study were affirmed by the published guideline of The Care and Use of Laboratory Animals (NIH Publication No. 85–23, revised 1996) and reported in accordance with ARRIVE guidelines, and approved by the Ethics committee of Tabriz University of Medical Sciences (IR.TBZMED.REC.1400.103) that complied with the Helsinki declaration.

## Supplementary Information

Supplementary Information.

## Data Availability

All data generated and/or analyzed during this study are included in this published article. The datasets used and/or analyzed during the current study are available from the corresponding author on reasonable request.
