# Supplementary material for: A novel injectable hydrogel containing polyetheretherketone for bone regeneration in the craniofacial region
Source: Sci Rep. 2023 Jan 17;13:864. doi: 10.1038/s41598-022-23708-6 (PMC9845302; doi:10.1038/s41598-022-23708-6)
Supplement: Supplementary file 1 — Supplementary Information. [file 41598_2022_23708_MOESM1_ESM.pdf]

SUPPLEMENTARY MATERIAL

Title Page:

A novel injectable hydrogel containing polyetheretherketone for bone regeneration in the craniofacial region

Mahdieh Alipour, DDS [1], Marjan Ghorbani, Ph.D. [2], Masume Johari khatoonabad, DDS, MSc [3], Marziyeh Aghazadeh DDS, MSc[4,5*].

[1] Dental and Periodontal Research Center, Faculty of Dentistry, Tabriz University of Medical Sciences, Tabriz, Iran.

[2] Nutrition Research Center, Tabriz University of Medical Sciences, Tabriz, Iran

[3] Department of Oral Radiology, Faculty of Dentistry, Tabriz University of Medical Sciences, Tabriz, Iran.

[4] Stem Cell Research Center, Tabriz University of Medical Sciences, Tabriz, Iran

Tabriz, Iran.

[5] Department of Oral Medicine, Faculty of Dentistry, Tabriz University of Medical Sciences, Tabriz, Iran.

[*] **Corresponding authors:** Dr. Marziyeh Aghazadeh, E-mail address: maghazadehbio@gmail.com.

Address: Daneshgah St, Golgasht St, Faculty of Dentistry, Tabriz University of Medical Sciences, Tabriz, Iran.

Postal code: 5166614711

A)

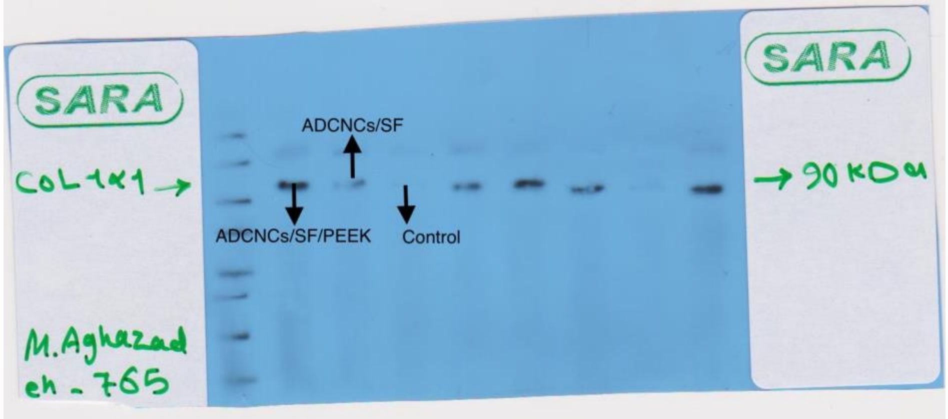
SARA
SARA
CoL1α1 →
→ 90 KDa
ADCNCs/SF
ADCNCs/SF/PEEK
Control
M.Aghazadeh - 765

B)

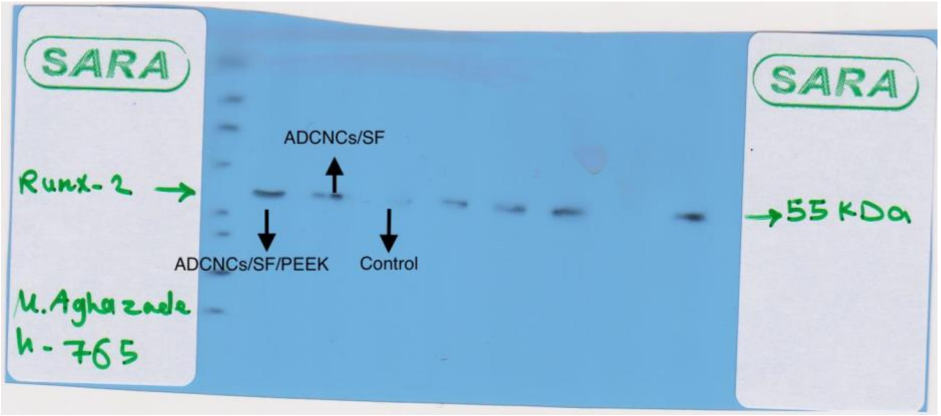
SARA
SARA
Runx-2 →
→ 55 KDa
ADCNCs/SF
ADCNCs/SF/PEEK
Control
M.Aghazadeh - 765

C)

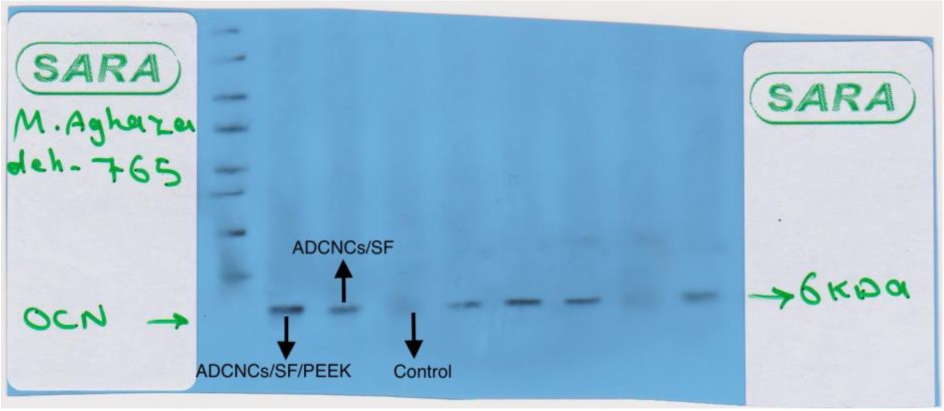
SARA
SARA
M.Aghazadeh - 765
OCN →
→ 6 KDa
ADCNCs/SF
ADCNCs/SF/PEEK
Control

D)

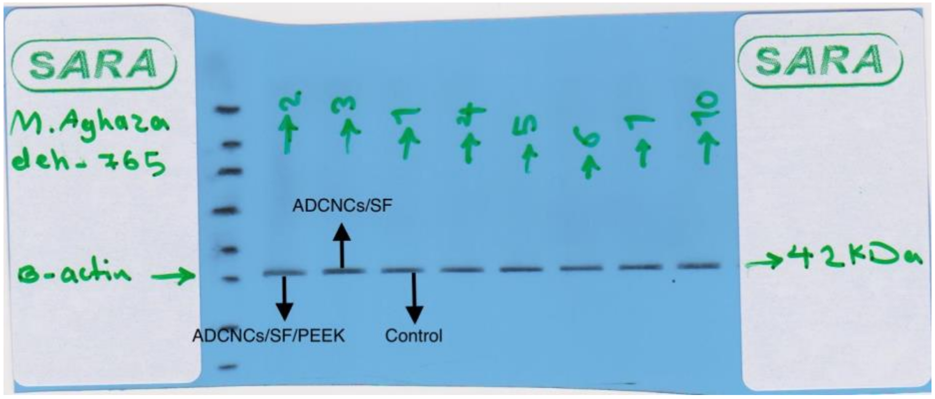

supplementary figure S1. Osteogenic differentiation of hDPSCs cultured in Aldehyde-cellulose nanocrystalline/silk fibroin (ADCNCs/SF) and ADCNCs/SF/PEEK Hydrogels. The human dental pulp stem cells are considered a control group. The cell lysates were prepared and used for western blot with A) Col1a1, B) RUNX2, C) OCN, D) B-actin. The gels were not cropped.
